# Association between Incidence of Prescriptions for Alzheimer’s Disease and Beta-Adrenoceptor Antagonists: A Prescription Sequence Symmetry Analysis

**DOI:** 10.3390/ph16121694

**Published:** 2023-12-06

**Authors:** Ali Alghamdi, Maarten J. Bijlsma, Stijn de Vos, Catharina C.M. Schuiling-Veninga, Jens H. J. Bos, Eelko Hak

**Affiliations:** 1 Groningen Research Institute of Pharmacy, Pharmaco Therapy, Epidemiology & Economics, University of Groningen, 9713 AV Groningen, The Netherlandss.de.vos@rug.nl (S.d.V.); c.c.m.schuiling-veninga@rug.nl (C.C.M.S.-V.); e.hak@rug.nl (E.H.); 2 Laboratory of Population Health, Max Planck Institute for Demographic Research, 18057 Rostock, Germany; 3 Department of Epidemiology, University Medical Center Groningen, University of Groningen, 9713 GZ Groningen, The Netherlands

**Keywords:** Alzheimer’s disease, beta-blockers, prescription sequence symmetry analysis, pharmacoepidemiology

## Abstract

Background: Alzheimer’s disease (AD) is the most common cause of dementia, with a growing number of patients worldwide. The association between AD and treatment with drugs targeting the beta-adrenergic receptor is controversial. The aim of this study is to assess the association between the initiation of AD medication and beta-adrenoceptor antagonists (beta-blockers) in adults. Materials and Methods: We conducted a prescription sequence symmetry analysis using the University of Groningen IADB.nl prescription database. We determined the order of the first prescription for treating AD and the first prescription for beta-blockers, with the dispensing date of the first prescription for AD defined as the index date. Participants were adults over 45 years old starting any AD medication and beta-blockers within two years. We calculated adjusted sequence ratios with corresponding 95% confidence intervals. Results: We identified 510 users of both AD and beta-blockers, and 145 participants were eligible. The results were compatible with either a significant decrease in the incidence of AD after using beta-blockers (adjusted sequence ratio (aSR) = 0.52; 95% CI: 0.35–0.72) or, conversely, an increase in beta-blockers after AD medication (aSR = 1.96; 95% CI: 1.61–2.30). Conclusions: There is a relationship between the use of beta-blockers and AD medications. Further research is needed with larger populations to determine whether drug therapy for AD increases the risk of hypertension or whether beta-blockers have potential protective properties against AD development.

## 1. Introduction

Alzheimer’s disease (AD) is a type of dementia, accounting for 50–60% of dementia cases [1]. More than 25 million people worldwide are affected, with 5 million new cases occurring each year [2]. In the US, an increase in the number of AD patients from 5.8 million in 2020 to 13.8 million in 2050 is expected. The disease is the fifth leading cause of death among older Americans, with a mortality rate increasing by 146% from 2000 to 2018 [3]. In Europe, 4.4% of older people have AD, while in the US, 9.7% of people over 70 years have AD [2].

According to the Dutch National Dementia Strategy 2021–2030, there are more than 280,000 people living with dementia, with about 178,000 currently registered in healthcare institutes. This number is expected to exceed half a million by 2040 [4].

AD is associated with two lesions: neurofibrillary tangles (NFTs) and senile plaques [5]. Overexpression of the amyloid precursor leads to amyloid-beta (Aβ) accumulation and formation in neuronal cells. An increase in phosphorylation of tau protein was found in AD patients, resulting in aggregation into NFTs [6]. Several symptoms are observed in AD patients, including dementia, memory loss, agitation, and an inability to perform daily tasks. Many risk factors contribute to the development of AD. The most common risk factor for AD is age, with an estimated prevalence of AD of 19% in people aged 75–84 and up to 50% in individuals over 85 years [7]. Other risk factors include gender (with females at higher risk than males), genetic variations (APP, PSEN1, PSEN2, and APOEε4 allele), lifestyle factors, and the presence of metabolic diseases like obesity, diabetes, hypertension, dyslipidemia, or depression [7,8].

Possible pharmacological treatment options include cholinesterase inhibitors (ChEIs) (donepezil, galantamine, and rivastigmine) and N-methyl-D-aspartate (NMD)-agonists like memantine. However, these treatments are not very effective and often have common side effects that prevent their use in many AD patients [9]. Importantly, these medications may have a detrimental effect on blood pressure. A study conducted to test acetylcholinesterase inhibition as a novel approach to treat neurogenic orthostatic hypotension concluded that these medications significantly increased orthostatic blood pressure and maintained elevated blood pressure [10]. Different case reports have also noticed an increase in blood pressure in patients admitted to hospitals with rivastigmine overdose [11,12].

Beta-adrenergic receptors belong to the superfamily of G-protein-coupled receptors, with three main types. They are distributed in different systems in the body, including the cardiovascular (CVS) and central nervous system (CNS) [13]. Several drugs act on adrenergic receptors, either blocking or activating them. For example, propranolol is a non-selective beta-adrenoceptor antagonist (beta-blocker) that blocks both beta-1 and beta-2 adrenergic receptors. The therapeutic use of propranolol is widespread, including the treatment of arrhythmia, myocardial infarction, and other conditions such as migraine and portal hypertension [14]. Beta-blockers (BBs) are the fourth most commonly prescribed antihypertensive drug class. In the UK, there has been an increase in the number of BB prescriptions from 2.6% to 8.6% between 1988 and 2018 [15]. In the US, 22.3% of patients aged 60–79 are using BBs for high blood pressure [16]. BBs are mostly lipophilic and can cross the blood–brain barrier (BBB). Highly lipophilic BBs, such as propranolol and timolol, diffuse rapidly through the BBB. Moderately lipophilic BBs (metoprolol, bisoprolol, carvedilol, and nebivolol) are less permeable. Lastly, hydrophilic BBs, such as atenolol and sotalol, are lacking this property [17].

There is controversy regarding the effect of BBs on the development of AD. Gliebus and Lippa conducted a study to investigate the influence of BBs on patients with cognitive impairment and found that patients on CNS-active beta-blockers (like propranolol) had worsened memory retrieval and lower Mini-Mental State Examination (MMSE) scores compared to patients not on CNS-active beta-blockers [18]. Another study investigated the effect of different BBB-permeable BBs on lowering the risk of AD. In a cohort of 69,081 people treated with BBs for hypertension, highly permeable BBs were associated with low risk of AD compared to lowly permeable BBs [19]. However, other studies suggest that antihypertensives decrease the risk of AD and also have a positive impact on cognitive impairment [20,21]. Medications like calcium channel blockers (CCBs) [22], angiotensin II receptor blockers (ARBs) [23], angiotensin-converting enzyme inhibitors (ACEIs) [24,25], and diuretics [25] have possible beneficial impacts on cognitive function in old people. Since epidemiological studies can be flawed due to confounding by indication, it remains unclear whether a potential causal relationship might exist between AD medications and blood pressure lowering by beta-antagonists. To investigate this further, we aimed to examine the association between the use of BBs and the use of AD medications.

To reduce confounding by design, we set out to estimate the association between starting BBs (e.g., propranolol) and starting AD drugs using prescription sequence symmetry analysis (PSSA). PSSA is a self-controlled observational design that takes between-person confounding into account. PSSA is a tool used for detecting adverse drug events using computerized claims data and is also employed in pharmacovigilance for identifying abrupt side effects (Lai et al., 2017) [26]. The tool was proposed by Hallas to identify associations between the use of cardiovascular medications and depression (Hallas, 1996) [27]. This method is used when a drug B (marker drug) is prescribed to treat an adverse event caused by drug A (index drug). The risk is then estimated by calculating the crude sequence ratio (cSR) by the number of participants initiating the index drug before the marker drug divided by the number of participants initiating the marker drug before the index drug. Adjusted sequence ratio (aSR) is then applied to account for a secular trend of prescribing the drugs over time [28].

## 2. Results

**Figure 1:**
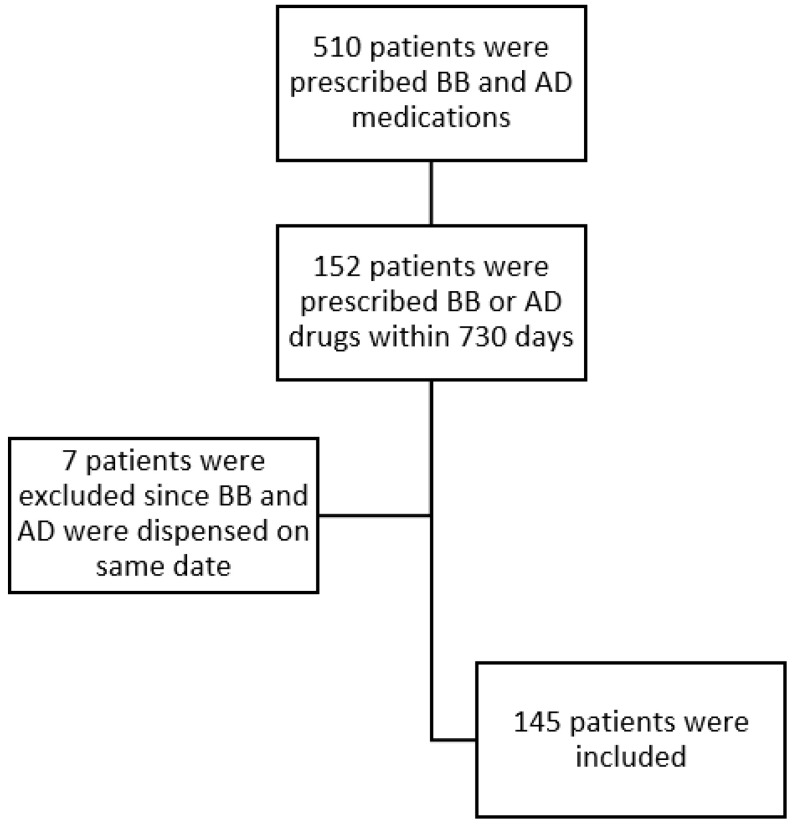
Flow diagram of the patients included in and excluded from the analysis.

The results indicated that 510 patients aged 45 years and older were prescribed both a BB and an AD medication. After excluding patients with a period longer than 730 days between the two medications, 152 patients could be included in the analysis. Finally, we excluded patients with dispensing of the BB and AD medication on the same date because it would be impossible to determine their prescribing order. In total, 145 patients were in the BB and AD group (see Figure 1).

Baseline characteristics revealed that 55% of the patients included in this study were males, while 45% were females, and the mean age was 75 (SD 8). The analysis of prescribed drug therapies showed various AD medications prescribed, with rivastigmine being the most common medication (71.7%), followed by galantamine (26.2%), memantine (1.4%), and then donepezil (<1%). The most commonly prescribed BB medications were metoprolol (62%), bisoprolol (21.4%), and propranolol (6.8%) (see Table 1).

The PSSA results for the group of patients starting both BB and AD drugs showed that the number of patients who used BBs first was 50, while the number of patients who used AD medications first was 95. In this scenario, the crude SR suggested a decrease in the number of patients starting AD medications after using BBs compared to patients using them before (cSR = 0.52, aSR = 0.52, 95% CI 0.35–0.72). The association was more pronounced for propranolol (cSR = 0.42, aSR = 0.14, 95% CI 0.036–0.53). If the scenario is reversed, the cSR and aSR for all BBs would be 1.9 (95% CI 1.61–2.3) (Table 2).

**Figure 2:**
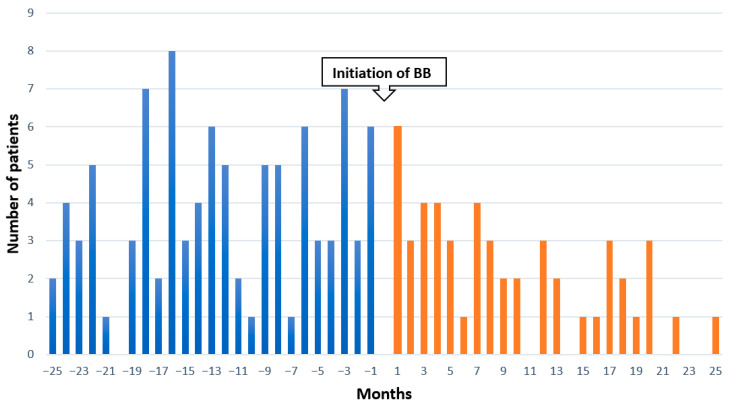
Graphical representation of the number of patients with the first prescription of the marker drug before and after the first prescription of the index drug. The blue bars represent the number of participants using AD medications before initiation of BBs while the orange ones are the participants using AD medications after initiation of BBs.

The graphical representation of the results displays the initiation of both drugs before and after within 24 months. The blue bars represent patients prescribed AD drugs before BB drugs, while the orange bars represent the opposite order. The number of patients with AD → BB is higher than BB → AD drugs (see Figure 2).

**Figure 3:**
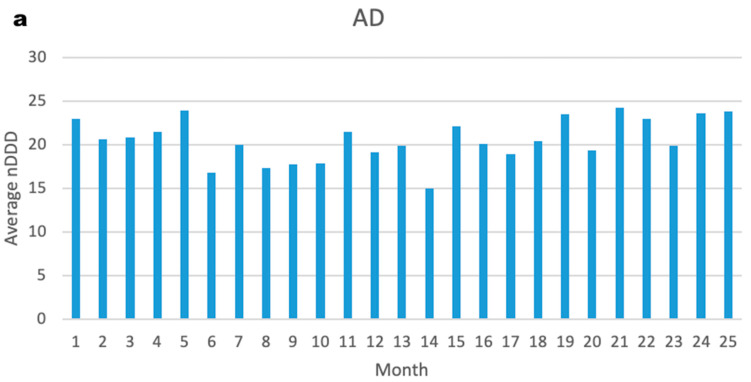

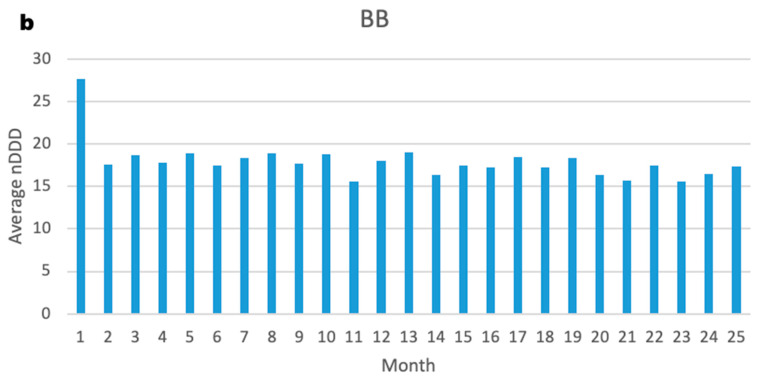
(**a**) The average number of defined daily doses of AD medications per patient per month. (**b**) The average number of defined daily dose of BB medications per patient per month.

After examining the trends in nDDD of drugs over time for the BB group, on average, there was no significant increase or decrease in the nDDD of both BBs and AD medications. This indicates that BBs had no substantial effect on the dose of AD medications, and vice versa (see Figure 3a,b).

## 3. Discussion

In total, the order ratios in this PSSA indicate either a potential preventive effect of BBs on starting AD medications or an increased risk of starting BBs after the initiation of AD medications. Although the total number of patients in the screening was only 145, both the crude and adjusted sequence ratios suggested a potential relationship between the use of BBs and AD medications.

The majority of AD patients in this study were on AChEIs (71.7% on rivastigmine and 26.2% on galantamine). According to European Federation of the Neurological Societies (EFNS) guidelines for AD management, AChEIs were considered the first-choice treatment at the time of diagnosis, with memantine suggested in cases of moderate to severe AD [29]. In our study, metoprolol was the most frequently prescribed BB agent (62%), followed by bisoprolol (21.4%) and then propranolol (6.8%). To support our findings, a study including 22,476 Dutch patients with cardiovascular diseases observed an increase in the number of prescriptions for BBs from 40% in 2001 to about 70% by 2015, with metoprolol being the most prescribed BB (>40%) followed by bisoprolol [30].

Several studies have concluded that there is a relationship between antihypertensives and the incidence of AD. According to Rouch et al., antihypertensive medications decrease the risk of developing AD, with eight out of eleven longitudinal studies showing a positive effect on AD [18]. The Honolulu-Asia Aging Study (HAAS) on antihypertensives also concluded that the use of BBs as monotherapy is associated with a lower risk of cognitive impairment [21]. A systematic review including 15 studies (*n* = 5879) investigated the association between hypertension and AD, with four studies showing a clear association between hypertension and AD neuropathological changes. The study concluded that hypertension may increase the risk of AD, while the use of antihypertensives could have a protective role against developing AD [31]. Peskind et al. conducted a placebo-controlled study to assess the effect of propranolol on patients with AD and found that propranolol treatment led to a moderate improvement in overall behavior [32].

Our findings could also be explained from a pharmacological perspective. One study reported that patients with AD have lower levels of beta-adrenergic receptors and lower beta-adrenergic receptor-stimulated cAMP [33]. Cyclic adenosine monophosphate regulates MAPK phosphorylation, which increases the concentration of Aβ and NFTs. When Aβ binds to beta-adrenergic receptors, it activates the cAMP/PKA signaling pathway, which increases the excitatory neurons leading to agitation, considered a symptom of AD. Hence, blocking the receptors with BBs may reduce agitation episodes [34,35].

Our findings also showed a potential effect of AD medications on BBs, with the number of BB prescriptions being higher after patients started using AD medications. A systematic review and meta-analysis study was conducted to evaluate the cardiovascular effects of acetylcholinesterase inhibitors in individuals with dementia. Thirty-one studies were analyzed, including 258,540 patients with dementia compared to over 2 million controls. After a median follow-up time (3–436 weeks), there was an association between ChEIs and hypertension in seven studies (*n* = 1573 patients, 4%, 95% CI = 2–8%, *p* < 0.001, I2 = 47%) [36].

Our findings support this possible association and may indicate substantial blood pressure effects immediately after the start of AD medications.

## 4. Method

### 4.1. Study Population

We included patients registered in IADB.nl (a prescription claims database) between 1994 and 2020. Patients aged 45 years or older at the index date were included. The index date is defined as the date of the first dispensing of any of the beta-blockers (see further). We took into consideration the use of other chronic medications (antihypertensives, antidiabetics, statins, thrombolytics, and anti-Parkinson’s drugs) as patient covariates at the index date.

### 4.2. Study Design and Data Source

In the PSSA method, the date of the first prescription of AD medications and BBs was determined for each patient. The first prescription of both types of medications that occurred within 730 days of each other for the same patient was included in the analysis. Prescription sequence symmetry analysis (PSSA) is a tool used in pharmacovigilance to detect adverse drug events using an extensive health record database [26,27,28].

We utilized the widely researched prescription database IADB.nl from the University of Groningen [37]. This database contains information about dispensed medications for over 25 years, encompassing more than 1,120,000 patients and over 120 community pharmacies. Registration in the database is independent of health care insurance, age, and gender. Prescription rates among the database population have been found to be representative of the Netherlands as a whole [38]. Throughout the database period, each person is followed individually, and prescription records include details on the date of dispensing, the amount dispensed, the dosage regimen, the number of days the prescription is valid, the prescribing doctor, and the drug (ATC code). Date of birth and gender are known, and each patient has a unique anonymous identifier. The medication records for each patient are comprehensive, with the exception of over-the-counter (OTC) medications and medications given out during hospitalization.

### 4.3. AD Drug Therapies

There are two classes of drugs approved by the FDA for treating AD. The first group contains cholinesterase inhibitors (donepezil; ATC code N06DA02, rivastigmine; ATC code N06DA03, galantamine; ATC code N06DA04). They are considered the first line of treatment for mild, moderate, and severe AD. The second group contains N-methyl D-aspartate (NMDA) antagonists. Most of the NMDA receptor antagonists failed due to severe side effects, and only memantine (ATC code N06DX01) was approved by the FDA to treat moderate to severe AD [9,39]. All patients in this study must have a first-use prescription for one of these drugs, meaning no history of these drug therapies for AD during at least 730 days before the start date. The first prescription was considered as the marker drug, while in the reverse analysis, the first prescription was considered as the index drug.

### 4.4. Beta-Blocker Drug Therapy

All patients in the study must have a first-use prescription of any BB, including propranolol (ATC C07AA05), celiprolol (ATC C07AB08), metoprolol (ATC C07AB02), bisoprolol (ATC C07AB07), sotalol (ATC C07AA07), atenolol (ATC C07AB03), carvedilol (ATC C07AG02), or nebivolol (ATC C07AB12). This means that there should be no history of BB medications at least 730 days before the starting date. The first prescription of BBs was considered as the index drug (also as the marker drug in the reverse analysis). Individuals who started AD medications and BBs (prescribed both medications) on the same day were excluded from the study because it is impossible to differentiate the order of prescriptions for such participants. We further calculated the average number of defined daily doses (nDDD) of BBs within 730 days of the first prescription of AD drugs, and we calculated the average nDDD of AD drugs within 730 days of the first prescription of BBs.

### 4.5. Outcome Associations

We estimated the association of BB drugs with the incidence of a first AD drug prescription.

First, the risk was estimated based on the cSR with the scenario that BBs precede AD drugs. It was calculated by dividing the number of patients prescribed the BB drug first followed by the AD drug second by the number of patients prescribed the drugs in reverse order.

cSR = Number of BB drug → AD drug/Number of AD drug → BB drug

If the cSR is less than 1, this indicates a possible decrease in the risk of an AD drug event due to a BB drug. If cSR is more than 1, then there is an increased risk of the AD drug event occurring. If the scenario is reversed, the AD drugs may prevent or increase the risk of starting BB drugs. Further, to overcome the potential bias by prescribing trends over time, we calculated the null-effect sequence ratio (neSR) (after calculating the overall average probability of AD drug to be prescribed after the BB drug (*Pa*)), which adjusts the ratio for the background rate of the medications under study. The neSR was calculated based on Hallas’ method [27]. The adjusted sequence ratio (aSR) was then calculated as aSR = cSR/neSR following the formula mentioned in [28].

$$P_{a}=\frac{\sum_{m=1}^{u}\left[I_{m}\times \left(\sum_{n=m+1}^{m+d}M_{n}\;\right)\right]}{\sum_{m=1}^{u}\left[I_{m}\times \left(\sum_{n=m-d}^{m-1}M_{n}+\sum_{n=m+1}^{m+d}M_{n}\right)\right]}$$

where:

*u* = last day of the study period;
*m* = a given day within the study period;
*I_m_* = number of incident users of the index drug on a given day;
*d* = specified number of days within the study period;
*n* = consecutive days of the study period (exposure window);
*M_n_* = number of patients receiving first marker drug on a given day.

In a descriptive analysis we examined the patterns of average nDDD of the marker drug before or after the index drug. We divided the total nDDD of AD medications by the number of patients per month.

## 5. Strengths and Limitations

This study used PSSA to estimate the association of initiating beta-blockers and initiating AD medications. The PSSA technique was advantageous because it includes patients with both drugs as a case-only design. Since PSSA is used to investigate associations, opposite associations can also be detected. Wahab et al. studied the validity of sequence symmetry analysis for adverse drug events. A total of 120 randomized placebo-controlled trials were reviewed for 19 medications to indicate adverse drug events using PSSA. The results showed that sequence symmetry analysis has moderate sensitivity (61%) and high specificity (93%) [40]. Another advantage of PSSA is its simplicity and minimal dataset requirements. A simulated PSSA was performed using 1000 scenarios for a population of 1 million patients on two different medications. The results of this study suggested that it may be a reliable tool to detect adverse drug events and also be useful for early detection of harm [41]. The limitation of this tool is time-varying confounding. A one-year window has been most frequently used to achieve good sensitivity and specificity [24]. Although, there is no standard exposure time window for PSSA [26], a two-year window in our study might increase the chances for time-varying confounders like disease progression and aging.

One of the limitations of this study is the relatively low number of patients included in the analysis. This could be due to the limited number of AD patients who used the medications. As mentioned earlier, in the Netherlands, there are over 280,000 people with AD, but according to Stichting Farmaceutische Kengetallen (SFK), only 30,000 Alzheimer’s disease patients were treated with AD medications in 2016, and the number is expected to decrease in the future. The reason could be the minimal effect of AD medications on the course of the disease [4,42]. This is why in this study, no stratification by age and sex was calculated. Further investigation is required with larger groups to validate the results. The database used in this study only included data on medications prescribed and dispensed for outpatients, so there are no data regarding drugs received during hospitalization. A study conducted on newly diagnosed AD patients versus patients with Lewy body dementia (LBD) found that over the first five years after diagnosis, 77% of the patients were hospitalized, and 73% of them had unplanned hospitalizations within the first year. The length of stay in the hospitals can reach up to 14 days per person per year for LBD and 7 days for AD patients. The reasons for the hospitalization differ due to comorbidity and polypharmacy [43]. Another limitation is due to the nature of the disease. One of the manifestations of AD is memory impairment, which may lead to poor medication adherence, eroding the observed associations, especially in the absence of a caregiver. According to Sönke Arlt et al., 16–40% of older adults with cognitive impairment forget to take their medications [44].

In conclusion, commonly prescribed AD medications appeared to increase the short-term risk of hypertension, rather than beta-blockers having a protective effect against developing AD in this short time window. Further studies are needed to validate our results with larger numbers of patients.

## Figures and Tables

**Table 1:** Drugs included in the study analysis and the prevalence of use at drug initiation.

Medication	Patients, *n* (%)
BB	
Metoprolol	90 (62)
Bisoprolol	31 (21.4)
Propranolol	10 (6.8)
Sotalol	7 (4.8)
Atenolol	3 (2)
Nebivolol	2 (1.4)
Carvedilol	1 (0.68)
Celiprolol	1 (0.68)
AD medications	
Rivastigmine	104 (71.7)
Galantamine	38 (26.2)
Memantine	2 (1.4)
Donepezil	1 (0.68)

**Table 2:** The outcome of PSSA of patients using BBs as index medications first against patients using AD medications first. The reverse association is also calculated.

	Total Number	BB Medication First	AD Medication First	cSR	neSR	aSR	95% CI
BB and AD medications	145	50	95	0.52	1.02	0.51	0.35–0.72
Reverse order for BB and AD medications	145	50	95	1.96	0.96	1.96	1.61–2.3

## Data Availability

The study protocol is available upon request by emailing the corresponding author.
